# A qualitative study on users’ perspective on the functioning of the National Non-Communicable disease portal in Dakshina Kannada district, Karnataka

**DOI:** 10.1186/s12913-026-14008-0

**Published:** 2026-01-19

**Authors:** N. S. Smrithi, Kumar Sumit, Sabah Mohd Zubair

**Affiliations:** 1 Department of Global Public Health Policy and Governance, Prasanna School of Public Health, Manipal Academy of Higher Education, Manipal, Karnataka 576104 India; 2 NP-NCD, District Health and Family Welfare Society, Mangalore, Karnataka 575001 India

**Keywords:** Digital health, Thematic analysis, Non-communicable diseases, National NCD portal

## Abstract

**Background:**

The Government of India recognizes the utilization of uniform information, communication, and technology (ICTs) applications which can improve understanding of the nation’s expanding health demands and rising non-communicable disease (NCD) burden. One such intervention is the development of the “National NCD Portal” was to aid healthcare providers in Population-Based Screening (PBS) initiative for common NCDs (diabetes, hypertension, cancers of the oral cavity, breast, and cervix) above 30 years of age. When such digital tools are introduced in healthcare, it is critical to understand users’ perspectives and experiences to further facilitate the increased integration of uniform ICTs into healthcare.

**Materials and methods:**

The study exhibits a qualitative exploratory design that involves a thematic analysis of 19 in-depth interviews of users (5 Accredited Social Health Activists, 4 Auxiliary Nurse Midwives, 5 Community Health Officers, and 5 Medical Officers) from 5 different taluks of the district selected by purposive sampling.

**Results:**

The users highly appreciated the user-friendly aspects of the portal, noting its positive impact on non-communicable disease management through enhanced accessibility of patient data to deliver better patient care. The key barriers were heavy workload, digital incompetence, and lack of accountability. The suggestions for the optimal utilization were the recruitment of a dedicated data entry operator and better accountability in addressing users’ needs.

**Conclusion:**

The National NCD portal has shown a change in enhancing the management of NCDs through better data management and accessibility. Addressing technical reliability, enhancing support systems, and ensuring adequate staffing are essential steps to optimize the portal’s functionality. The study results recommend future evaluations in various settings to understand the long-term impact of the portal or any digital health interventions.

**Supplementary Information:**

The online version contains supplementary material available at 10.1186/s12913-026-14008-0.

## Introduction

Non-communicable diseases (NCDs) are the major cause of death globally, accounting for 74% of all deaths [1]. In India, NCDs contribute 63% of total deaths, reflecting a significant public health concern. The transition from infectious diseases to NCDs poses a threat not only to individual health but also to the socio-economic development of the nation. In response, the Government of India has initiated various strategies, including the implementation of digital health interventions to manage and mitigate the rising burden of NCDs [2].

In collaboration, with the Ministry of Health and Family Welfare, Tata Trust, and Dell technologies, software has been developed, the National NCD Portal for healthcare providers including from the root level to assess the degree of population-based screening initiative for the five common NCDs (diabetes, hypertension, oral, cervix and breast cancers), implementation and easy gathering of data of the NCDs at National, State, District, Block, Primary Health Care Center (PHC) and Sub-Health Center (SHC) levels. The National NCD Portal manages the follow-up and management of individuals diagnosed with any of the five NCDs. It is offered as a tablet or mobile-based application for ASHA/ANM and web-based software for PHCs, Community Health Centers (CHC), Taluk and District hospitals (DH) level facilities [3].

The portal is integrated with the Ayushman Bharat Digital Mission (ABDM) for the generation of Ayushman Bharat Health Account (ABHA), a self-declared login, acts as a unique ID for sharing and digital access to medical records and to update NCD facility data with HWC information and to input NCD indicators into the HWC Service Delivery Format, the National NCD Portal is also integrated with the Ayushman Bharat Health and Wellness Centre Portal (AB-HWC) [4]. Currently, 31 states and union territories are utilizing the portal, one state is onboarding in progress, and four states and union territories are sending aggregate data through Application Programming Interface (API). Also, through the portal 53.94 crore beneficiaries have been enrolled, 8.48 crore ABHA members generated and 3.95 crore patients are under treatment for hypertension and diabetes [3, 4].

Over the years, since 2000, the burden of NCDs in Karnataka has overtaken that of infectious diseases, coming in at over 62% [5]. To address the issue, the NCD program established PBS in the state in 2020 and to improve the initiative execution and service delivery, the Tata Trusts intervened and leveraged information technology by utilizing a full Comprehensive Primary Health Care (CPHC) application integrated with National NCD portal to support the PBS initiative in Karnataka. The assessment showed enrolment numbers went up and the state improved from eighth place in October 2020 to second place by March 2021 on the national dashboard [6]. With the help of mobile applications, healthcare providers may better organize their time and deliver higher-quality management of patient care [7]. However, it is equally important to understand the user experience, which aids in the continuous improvement and effective utilization of digital intervention in the future. Understanding how users interact with these technologies, their challenges, and their needs can lead to more efficient and impactful healthcare solutions. Understanding the perspectives of frontline users is critical to the successful adoption and sustainability of digital health interventions. Previous studies highlight that overlooking user experiences often results in underutilization or failure of such systems, particularly in LMIC settings where contextual barriers are significant [8–11]. Capturing district-level user insights is, therefore, essential to ensure that platforms like the NCD portal are not only technically sound but also acceptable, feasible, and responsive to ground realities. The present manuscript gathers the users’ perceptions of the functioning of the National NCD Portal at the district level, Dakshina Kannada district, Karnataka.

## Materials and methods

### Study design and setting

This qualitative exploratory study was conducted in Dakshina Kannada, a southern coastal district of Karnataka. The participants included in the study were from Mangalore, Bantwal, Belthangady, Puttur, and Sullya which are the five taluks in Dakshina Kannada having 430 sub-centres, 77 PHCs, 8 CHCs, 4 sub-divisional hospitals, and 2 district hospitals [12]. This study was conceived as an exploratory qualitative inquiry, designed to capture rich, contextualised accounts of user experiences and operational challenges during the early phase of National NCD Portal implementation. Given this scope, we did not include quantitative measures of portal effectiveness or direct patient health outcomes, which will be essential to address in future mixed-methods evaluations. Noteworthy to mention, the research was conducted during the initial implementation phase, which limited the ability to evaluate long-term sustainability or outcomes.

### Study participants and sampling

The duration of the study was from January 2024 to June 2024. The purposive sampling technique was adopted in the current study to identify the study participants. An in-depth interview guide was developed, which covered the following domains: General questions, Knowledge, User experience, Training, Resources, Documentation, Challenges, and Suggestions. Semi-structured interviews were conducted primarily in the local language (Kannada) by the principal investigator under the supervision of the other two co-investigators. The transcripts were subsequently translated into English by professional translators, and a sample of translations was cross-checked by the research team for accuracy and cultural relevance. Participants were exclusively healthcare providers who operate the portal and are responsible for patient data entry and follow-up. While this group offers valuable operational insights, future studies should incorporate patient and community perspectives to assess usability, accessibility, and perceived benefits from the end-user standpoint. A total of 19 interviews were conducted (5 Medical Officers, 5 CHOs, 4 ANMS and 5 ASHAs) (See Table 1) and every interview lasted for about half an hour to forty minutes and was recorded with consent. Participants were contacted via telephone prior to the interview. Written informed consent was obtained from all participants prior to the commencement of the interviews. A participant information sheet detailing the study’s objectives, confidentiality safeguards, and the voluntary nature of participation was shared with each respondent. The interviews were conducted at their workplace, and no other person was present during the course of the interview.

**Table 1:** Participant characteristics

S.No	Cadre	Number of participants
1	ASHA’S	5
2	ANMs	4
3	Community Health officers	5
4	Medical Officers	5

### Data analysis

**Fig. 1:**
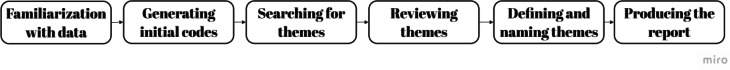
Six-step guide to good thematic analysis; Source-Braun & Clarke (2006) [13]

**Fig. 2:**
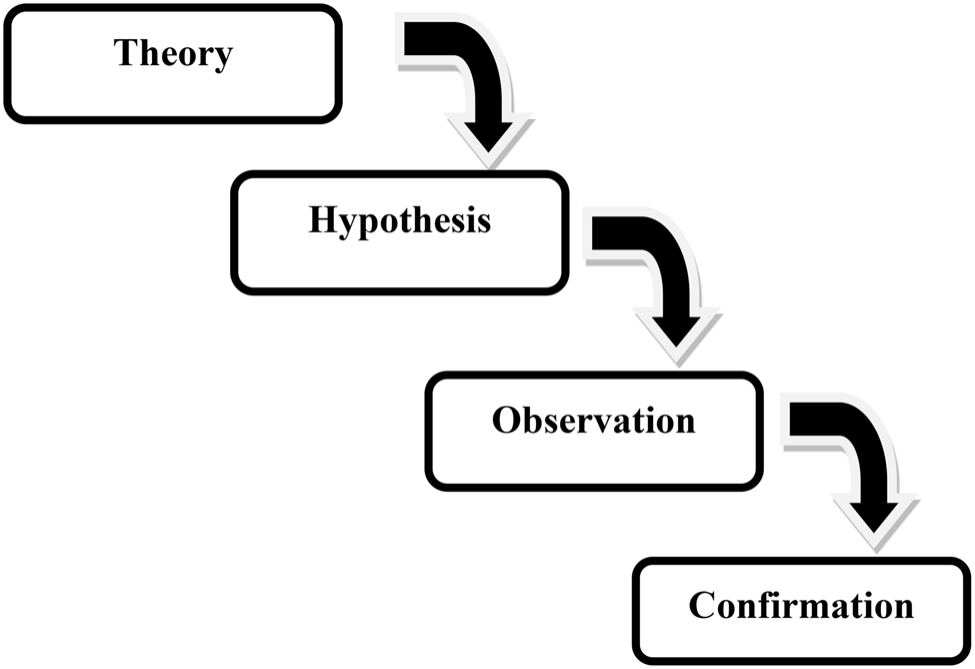
Deductive reasoning

Once the data set, was well organized and transcribed, the transcripts was then read through and over a number of times in order to become more familiar with the information gathered. Then relevant segment of the transcripts was coded according to the study objective [13]. These coded transcripts were then exported to ATLAS.ti (Version 24.1.0) software for the final analysis of the study. The categories and coded clusters using the research questions identified led to the development of broader themes as highlighted below. The emergent themes were iteratively developed from the coded data and were subsequently reviewed in relation to the research questions and the conceptual framework to ensure coherence and provide a meaningful interpretation of the findings. Thematic analysis was conducted following the process outlined by Braun and Clarke (Fig. 1) to identify, analyse, and report themes within the data. To further explain, the coded clusters and categories from the research questions guided the creation of more encompassing themes, as explained below. The codes were created iteratively, starting from open coding of the field notes and transcripts. Two members of the research team independently coded a subsample of the transcripts to produce a first codebook. Coding differences were resolved by consensus discussion, and the cleaned codebook was subsequently applied to the complete dataset. The transcripts were coded independently by the principal investigator and one co-investigator using ATLAS.ti software. Inter-coder reliability was assessed by double-coding, 20% of the transcripts, and discrepancies were resolved through consensus discussions to ensure consistency and credibility of the analysis.Thematic analysis is based on the identification of patterns through careful reading of the data and thematic structuring into categories. In thematic analysis, patterns were identified in a bottom-up procedure i.e., deductive reasoning. The method was data-driven and goes beyond semantic content, focusing on the latent level information, i.e., underlying ideas and concepts. The method is based on the researcher’s judgement rather than on quantifiable measures (Fig. 2). Furthermore, in addition to interview transcripts, the Principal Investigator maintained detailed field notes during data collection. These observations captured contextual information, including clinic workflow, staffing constraints, interruptions due to patient load, infrastructural limitations, and participants’ nonverbal responses. The observational data were used to contextualize participant accounts and were integrated during the coding and interpretation phases as part of methodological triangulation, thereby enhancing the credibility and depth of the analysis.

In order to increase the validity of the analysis, we utilized methodological triangulation by using a variety of data sources (e.g., interviews, documents, and field observation) to cross-check findings. Investigator triangulation was also ensured, since more than one researcher coded, developed the themes, and interpreted them. The emergent themes were constantly checked against the research questions and conceptual framework for consistency and theoretical congruence. These procedures provided a sound foundation for the findings’ credibility and trustworthiness, which are presented below in the broader themes.

### Ethics

The ethical approval was obtained from the Institutional Ethical Committee- Kasturba Medical College-Kasturba Hospital Institutional Ethical Committee (Reference number- IEC2/675/2023). All the participants involved in the study were well explained about the study objective and their written consent was obtained before every interview.

### Results

The thematic analysis of the interviews revealed several key themes related to the experiences of healthcare providers using the National NCD Portal (See Table 2). These themes are categorized into four main areas: User experiences, Barriers, the Impact on healthcare delivery, and suggestions.

**Table 2:** Themes, categories and codes

Themes	Categories	Codes
	(1.1) User-friendly	Ease of use
		Portability
(1) User Experience		Customization options
	(1.2) Training	Satisfactory training sessions
		Less training opportunities
		Post-training confidence
	(2.1) Data management	Accurate data
		Patient information accessibility
		Quality checks by officials
(2) Impacts on healthcare		
practices and outcomes		
	(2.2) Service enhancement	Improved NCD detection
		Improved health awareness
		Delivery of health services
	(3.1) Work overload	Data entry burden.
		Technical Interruption
		Time-consuming
		Staffing shortages
(3) Barriers	(3.2) Digital incompetence	Educational qualification
		Incorrect data entry
	(3.3) Less accountability	Less assistance
		Understanding staffing needs
	(4.1) Inadequate staffing	(4.1) Inadequate staffing
(4) Suggestions for further		
improvisations		
	(4.2) Accountability	Server problem fixation
		Adequate training for freshers
		Resource allocation
		Task eligible candidates

### User experiences

Most participants reported that the National NCD Portal improved patient data management and streamlined NCD screening and follow-up processes. They found the portal and its apps user-friendly, appreciating the portability that allowed access to patient data from anywhere via smartphones, reducing the need for carrying multiple registers. The software’s customization features made it easy to organize, edit, and retrieve patient information, enhancing data quality and minimizing errors. While most participants valued the multiple training sessions they received, some newer users with less experience felt they needed more comprehensive training to fully utilize the portal.

*It is easy to access and use. Through a link we can go to the portal with our respective numbers provided. Also*,* if I have to edit the already entered data*,* we can do that without much difficulty. (P13*,* MO 4)*.

*Regarding the use of this app*,* I only received training once. However, it was not useful for me because when I started using the NCD portal had some doubts and confusion regarding some issues (P7*,* ANM 2)*.

### Impact on healthcare practices and outcomes

The National NCD Portal has aided Medical Officers to access patients’ medical histories, especially when patients forgot their documents, making patient care decisions challenging. With the portal, patients receive individual IDs, allowing healthcare professionals to easily retrieve complete medical histories by entering the patient’s ID, even without physical files. This has improved data accuracy and reliability, with regular quality checks boosting user trust. The preference for soft copies over hard copies reflects a shift towards digital health, which is also more sustainable.

*Details of a particular patient can be derived from this app. This app helped us to maintain all the above details in one platform which was easily accessible. We appreciate the initiative taken by the Government to introduce this app. (P8*,* ASHA 2)*.

*I prefer maintaining soft copies over hard copies. (P9*,* MO 3)*.

The portal’s integration with the PBS initiative has increased NCD detection and health awareness, particularly through mandatory risk assessments conducted by ASHAs. This early detection enables timely intervention and treatment. The portal’s integration with the ABDM and ABHA health IDs allows users to track and access digital health records, lab reports, and prescriptions, enhancing healthcare delivery and empowering both providers and patients.

*This new initiative is very good to our society because we can know the health issues of people at the community and those health issues can be treated in the early stages. Also*,* we will know how cases are coming up*,* and is it increasing or decreasing. (P3*,* ANM 1)*.

### Barriers

Most participants identified workload issues due to inadequate staffing across all levels of the healthcare system. Medical officers found data entry difficult during busy clinic hours, often postponing tasks or seeking help, which increased everyone’s workload. Managing multiple portals, like the NCD and RCH programs, is time-consuming and exhausting. Field observations further indicated that interviews were often conducted amid ongoing clinical duties, with frequent interruptions due to patient care responsibilities, highlighting the extent of workload pressure described by participants.

*We lack staff*,* like badly*,* because it becomes difficult for the OPD patients and administrative work at the same*,* which affects the quality of work. (P13*,* MO 4)*.

*We have 9 sub-centers and only 4 staff*,* each person will get 2–3 sub-centers*,* how the work pressure will be. (P11*,* ANM 3)*.

Technical problems such as server crashes and poor internet connectivity, especially in rural areas, frequently interrupt work, forcing manual data entry that adds to the workload and stress. Despite the push for digital records, participants are still required to maintain physical copies, leading to double data entry and reduced productivity. Training effectiveness varied due to participants’ diverse educational backgrounds which led them to struggle with digital tasks. The PI also observed intermittent internet connectivity and delays in portal access at several facilities, which corroborated participants’ reports of server-related disruptions and reliance on manual documentation.

*Especially in rural areas due to poor network connection our work became even slower. (P12*,* ASHA 3)*

*Also maintaining paper records continues*,* nobody has ever asked to stop that which is too much of work for all other national programs to manage with the existing staff. (P9*,* MO 3)*.

*I could not understand the portal because I studied only till 8th standard. Even though we got training*,* it was difficult for me to understand and operate on my own. (P16*,* ASHA 4)*.

Most participants also reported inadequate technical support and poor-quality tablets, leading many to use personal devices or purchase new phones. Despite repeatedly requesting more assistance and staff, little action was taken, leaving them overwhelmed and unable to focus on primary healthcare duties effectively.

*I did not receive any sort of devices for the task. Our mobile phones were hanging*,* and we could not upload data. (P4*,* ASHA 1)*.

### Suggestions for improvement

Most participants unanimously agreed on the need for a dedicated data entry operator at every PHC. The current system burdens healthcare providers with both patient care and data entry, leading to stress and inefficiency. Hiring dedicated operators would reduce errors, ensure timely updates, and allow healthcare staff to focus on their primary responsibilities.

*Ideally*,* we need more dedicated staff for the data entry like data entry operator*,* worth it as it will ensure the data quality. Nowadays I am getting the work done with the help of pharmacists*,* and lab technicians. (P1*,* MO 1)*.

While participants generally viewed the training sessions as helpful, the study did not systematically collect information on their frequency, duration, content, or evaluation. Future assessments should examine the quality and comprehensiveness of training programmes and their influence on portal usage proficiency. Participants highlighted several areas for improving the NCD Portal. They emphasized the need for better server infrastructure to minimize downtime and technical issues, along with more training sessions for staff, particularly new recruits and periodic refreshers to keep users updated. Optimal resource allocation tailored to the specific needs of each PHC was also recommended, ensuring smooth operations across different settings. Additionally, participants stressed the importance of careful selection and training of candidates for data management tasks to enhance system performance and reduce errors.

*An adequate amount of training should be given to us. This is because among our staff some are only SSLC passed*,* and not all of us can use the app without any training. (P7*,* ANM 2)*.

*Improve the server problem*,* if the server is fast*,* the work also gets done. (P2*,* CHO 1)*.

## Discussion

The current study’s primary goal is to explore the perceptions of district-level users on the functioning of the National NCD portal in Dakshina Kannada, Karnataka. A scoping review conducted by Xiong et al.found [8] that there is a lack in the number of qualitative studies that assess local contexts and requirements prior to adopting digital health interventions in the primary healthcare settings of Low Middle-Income Countries (LMICs). The present manuscript highlights the extent the portal benefited the PBS initiative for the five NCDs (diabetes, hypertension, cancers of the oral cavity,breast, and cervix) through improved data collection and accessibility and has explored the barriers and user suggestions for further improvisation in the future. This study focused on micro-level operational experiences rather than macro-level determinants such as funding allocations, state-level ICT policies, or broader health system infrastructure. Integrating these organisational and policy perspectives in future evaluations would yield a more holistic understanding of portal implementation. Furthermore, given that some participants reported difficulty using the portal due to limited ICT skills or lower literacy levels, design adaptations such as simplified interfaces, icon-based navigation, multilingual support, and audio-guided instructions could improve usability for diverse user groups.

The findings collected from Dakshina Kannada yield important lessons for the future national scale-up of the NCD portal. The district’s relatively high degree of digital preparedness, founded on a pervasive culture of electronic reporting and trained health workforce, facilitated the introduction of the portal and reflects the benefits of leveraging existing digital infrastructure and human capacity. At the same time, the portal was considered a means to increase access to data and enable real-time monitoring, thereby enhancing the efficiency of screening and follow-up processes for diabetes, hypertension, and three priority cancers. This indicated that equivalent gains could be achieved on a larger scale with proper training and support. However, challenges such as connectivity problems, login delays, increased workload in data entry, and a lack of user-friendliness in the interface reflect systemic issues that are likely to emerge in other districts. Solutions to these problems through simplified data entry processes, offline functionality, and integration with existing health information systems will be critical to increased uptake. User recommendations for refresher training, supportive supervision, and feedback systems, coupled with technical improvements, further affirm the importance of user-centered design and workforce support in driving sustainable implementation. Taken together, these district-level findings yield transferable lessons that can guide the quality improvement and successful scale-up of the NCD portal across different Indian states.

The participants in the present study reported that the portal is generally easy to handle for efficient data entry and retrieval. They also agreed that the training sessions provided to them were helpful to a certain extent. The study also found that users value the portability of the portal, as it can be accessed via smartphones and tablets, enabling them to manage their tasks from various locations unless the user has a proper internet connection. The portal also offers customization options which the users think to be very helpful. It allowed them to organize and edit patient data whenever required, and update information with minimal effort, which also helped in reducing the errors and ultimately improving the data quality. A review study done by Jakob et al. [10] reported that the factors that showed positive effects on adherence across all health domains for continuous use of the mHealth app especially for NCDs are user-friendly and stable app design, reminders in the form of personalized push notifications, personalization or tailoring the content of mHealth apps to the unique needs of the user. Overall, the present study indicates that while the portal is largely user-friendly and has been effectively integrated into users’ workflows, there are areas where enhancements could make it even more accessible and efficient.

The study results show that the portal has significantly contributed to and enabled healthcare providers to collect, store, and access patient data more efficiently. This has facilitated better tracking of patient histories, medication adherence, and overall disease management, critical components in effectively treating and controlling NCDs. A review study conducted by Aizaz et al. [9] has indicated the use of EHR led to improved continuity of care, the early detection and treatment of NCDs, and the storage and exchange of patient data. The National NCD portal has empowered healthcare providers to access comprehensive and accurate health data which has aided them to deliver better healthcare services, make informed decisions, and provide more personalized care to their patients. ASHA workers reported that this has been instrumental in educating individuals about the risks and symptoms of NCDs, particularly cancers, leading to increased health awareness and proactive health behaviours among community members.

However, the users have expressed their needs in several areas which require improvement. Participants have conveyed issues related to the reliability of the technology, including frequent server problems and inadequate technical support, which have hindered their ability to use the portal effectively. Additionally, they have highlighted staffing concerns and needs. The existing healthcare providers are often overwhelmed with the dual responsibilities of providing patient care and managing data entry tasks for the NCD portal. The findings of this study share few similarities with the findings of the study conducted by Shilpa et al. [7] concerning the CPHM app for RCH services in Southern Karnataka which has also revealed barriers like the dual burden of data entry both in the app and mandatory registers and technical difficulties like poor internet connection faced in the field for data entry, especially in rural areas. Particularly, ASHAs and ANMs involved in this study expressed that not all the ASHAs and ANMs are well adapted to using smartphones and they are preoccupied with many other duties, with respect to other national health-based programs. Hence, they have suggested that when such technology is being introduced to them, the officials have to know who is eligible for the tasks to perform, select them accordingly, and provide them adequate support through multiple training sessions and resources, according to a review study conducted by Pati et al. [11] has reported that ASHAs and ANMS nationwide have not been given adequate training regarding NCD care and ASHAs are overburdened with many responsibilities assigned to them, like they are expected to act as educators, link workers, service providers, and activists. Hence, participants in the current study have highlighted the need for dedicated staff, such as data entry operators, who they believe/can help them reduce this burden, allowing healthcare providers to focus more on their primary responsibilities and improve the quality of care. Nevertheless, to address persistent connectivity and server downtime issues, technical enhancements could include offline data entry modes with subsequent synchronization, upgrading server infrastructure, implementing load-balancing systems, and developing mobile application versions of the portal to improve accessibility in low-connectivity settings.

### Limitations

First, the study relied solely on qualitative interviews, without incorporating quantitative indicators such as screening coverage rates, follow-up adherence, or NCD control outcomes. Future research should integrate such measures to provide a more comprehensive assessment of the portal’s effectiveness. Second, the study was restricted to Dakshina Kannada district due to feasibility considerations. Comparative studies across multiple districts or states could help identify context-specific facilitators and barriers, thereby improving the generalisability of findings. Third, the absence of patient-level data limits the ability to evaluate the portal’s perceived impact among beneficiaries. Including patient surveys or interviews in future evaluations would enhance understanding of its usability and effectiveness from the end-user perspective. Fourth, the study assessed short-term user experiences without applying a structured evaluation framework to determine success or failure. Longitudinal studies using established models such as RE-AIM (Reach, Effectiveness, Adoption, Implementation, and Maintenance) or the Technology Acceptance Model could provide robust insights into sustainability and scalability. Fifth, the study involved 19 participants from a single district, which restricts statistical generalisability. Nevertheless, the findings may offer transferable lessons for similar contexts.

## Conclusion

In summary, while the National NCD portal has shown a change in enhancing the management of NCDs through better data management and accessibility, there are important areas that require attention and improvement. Addressing technical reliability, enhancing support systems, and ensuring adequate staffing are essential steps to optimize the portal’s functionality and overall impact on healthcare practices and outcomes in Dakshina Kannada, Karnataka.

## Supplementary Information

Below is the link to the electronic supplementary material.

Supplementary Material 1

## Data Availability

No datasets were generated or analysed during the current study.
