# Supplementary material for: A qualitative study on users’ perspective on the functioning of the National Non-Communicable disease portal in Dakshina Kannada district, Karnataka
Source: BMC Health Serv Res. 2026 Jan 19;26:104. doi: 10.1186/s12913-026-14008-0 (PMC12829281; doi:10.1186/s12913-026-14008-0)
Supplement: Supplementary file 1 — Supplementary Material 1 [file 12913_2026_14008_MOESM1_ESM.pdf]

Study tool- Questionnaire

Topic: "A study on user's perspective on the functioning of National Non-Communicable Disease Portal in Dakshina Kannada District, Karnataka."

QUESTIONNAIRE

1) General questions:

1.1 Designation

1.2 How long have you been working at this centre?

1.3 Is your position permanent or contractual?

2) Knowledge:

2.1 Do you know about the National NCD portal?

2.2 Could you let me know in detail how you came to know about it?

2.3 What are the activities that can be done through the portal?

3) User Experience:

3.1 Is the software user-friendly?

3.1.1 If yes, please describe.

3.1.2 If no, what improvements, if any, would you suggest enhancing the user

3.1.3 What is your experience in using the National Non-Communicable Disease portal?

4) Community Impact:

4.1 Has the portal impacted the overall population-based screening initiative of NP-NCD in Dakshina Kannada District?

4.1.1 If yes please explain.

4.1.2 If no please explain.

4.2 How well the community cooperates in providing the details and documents to be entered into the app?

5) Documentation:

5.1 Do you prefer data entering using handheld devices over data entry using paper?

-If yes, please explain

5.2 Using handheld devices is it time-consuming and if so why?

5.3 Do you have to maintain multiple registers for it and why?

6) Training

6.1 How are the staff trained to use the portal?

6.1.1 How frequently the training sessions are conducted and how many per batch?

6.1.2 Are the training sessions adequate for different levels of staff?

7) Challenges

7.1 What are the challenges that you have encountered in the usage of the portal, please explain.

8) Suggestions and Opinions:

8.1 What is your opinion about the existing functioning of the portal?

8.2 Would you like to give any suggestions for any improvement?

Kumar Sumit
19th Dec. 2023

Name and Signature of Guide
Date: 19/12/23

Dr. Sabah Mohd Zubair

Name and Signature of Co-guide
Date: 19/12/23

Validated
Narayanan
19/1/2023
Dr. Prakash Narayanan
Professor, Dept. of Health Policy
PSPH, Manipal

PRASANNA SCHOOL OF PUBLIC HEALTH
MAHE MANIPAL
